# Predicting sepsis onset using a machine learned causal probabilistic network algorithm based on electronic health records data

**DOI:** 10.1038/s41598-023-38858-4

**Published:** 2023-07-20

**Authors:** John Karlsson Valik, Logan Ward, Hideyuki Tanushi, Anders F. Johansson, Anna Färnert, Mads Lause Mogensen, Brian W. Pickering, Vitaly Herasevich, Hercules Dalianis, Aron Henriksson, Pontus Nauclér

**Affiliations:** 1 Division of Infectious Diseases, Department of Medicine, Karolinska Institutet, Solna, Stockholm, Sweden; 2 Department of Infectious Diseases, Karolinska University Hospital, Stockholm, Sweden; 3 Treat Systems ApS, Aalborg, Denmark; 4 Department of Health Science and Technology, Center for Model-Based Medical Decision Support, Aalborg University, Aalborg, Denmark; 5 Department of Clinical Microbiology and the Laboratory for Molecular Infection Medicine (MIMS), Umeå University, Umeå, Sweden; 6 Department of Anesthesiology and Perioperative Medicine, Mayo Clinic, Rochester, MN USA; 7 Department of Computer and Systems Sciences, Stockholm University, Stockholm, Sweden

**Keywords:** Infectious diseases, Predictive medicine

## Abstract

Sepsis is a leading cause of mortality and early identification improves survival. With increasing digitalization of health care data automated sepsis prediction models hold promise to aid in prompt recognition. Most previous studies have focused on the intensive care unit (ICU) setting. Yet only a small proportion of sepsis develops in the ICU and there is an apparent clinical benefit to identify patients earlier in the disease trajectory. In this cohort of 82,852 hospital admissions and 8038 sepsis episodes classified according to the Sepsis-3 criteria, we demonstrate that a machine learned score can predict sepsis onset within 48 h using sparse routine electronic health record data outside the ICU. Our score was based on a causal probabilistic network model—SepsisFinder—which has similarities with clinical reasoning. A prediction was generated hourly on all admissions, providing a new variable was registered. Compared to the National Early Warning Score (NEWS2), which is an established method to identify sepsis, the SepsisFinder triggered earlier and had a higher area under receiver operating characteristic curve (AUROC) (0.950 vs. 0.872), as well as area under precision-recall curve (APR) (0.189 vs. 0.149). A machine learning comparator based on a gradient-boosting decision tree model had similar AUROC (0.949) and higher APR (0.239) than SepsisFinder but triggered later than both NEWS2 and SepsisFinder. The precision of SepsisFinder increased if screening was restricted to the earlier admission period and in episodes with bloodstream infection. Furthermore, the SepsisFinder signaled median 5.5 h prior to antibiotic administration. Identifying a high-risk population with this method could be used to tailor clinical interventions and improve patient care.

## Introduction

Sepsis is a severe organ dysfunction triggered by infections, and a leading cause of hospital admission and death. It is estimated to affect approximately 50 million patients and result in 11million deaths globally per year^1^. In sepsis, early antimicrobial treatment is key for survival, warranting structured approaches to guarantee timely identification^2–4^. The Surviving Sepsis Campaign Guidelines recommend hospitals to have sepsis screening for all acutely ill, high-risk patients^5^. Commonly used early warning scores to detect patient deterioration, such as National Early Warning Score (NEWS2), have a broader purpose and are not specifically developed for sepsis^6^. In many hospitals, electronic health records (EHR) are the leading communication platform in clinical work and contain temporal information on risk factors, vital parameters, and laboratory data^7^. The main challenge, however, is to use this information efficiently.

Machine learning models have gained increasing interest due to their ability to make predictions based on large amounts of data. Although the models per se are not novel, the advances in processor speed and digitalization of healthcare data have boosted the field^8^. Studies on machine learning models for sepsis screening have mainly focused on the intensive care unit (ICU) setting, and most have not been evaluated in a way that is appropriate to assess the clinical utility^9–11^. As an example, Henry et al. reported an area under receiver operating characteristics curve (AUROC) using one single screening point per patient, i.e., whether a given threshold was crossed at any point in time prior to septic shock^12^. However, this meant the positive screening could be unrelated in time to the sepsis onset and a substantial portion of patients in their study triggered more than 5 days before sepsis. Other studies have not clearly stated how the AUROC was calculated, making assessment of clinical utility difficult^13^. There is also substantial inconsistency between sepsis definitions and evaluation metrics, which makes direct comparison of studies complicated^14^.

The aim with this study was to develop and assess an automated sepsis prediction model outside the ICU, using data from EHR, and to evaluate the score in a clinically realistic use-case with comparison to conventional screening methods.

## Results

### Patient characteristics

The cohort included 82,852 hospital admissions of 55,655 patients. Median age was 63 years and 52.7% were women (Table 1). In total, 8038 (9.7%) sepsis episodes were identified, of whom 6889 (8.3%) where classified as community-onset (CO) and 1149 (1.4%) where classified as hospital-onset (HO). Sepsis patients had a median baseline Sequential Organ Failure Assessment (SOFA) score of 0 (interquartile range [IQR]: 0–1), at sepsis onset had a median SOFA of 2 (IQR: 2–4) and had a median worst SOFA of 3 (IQR: 2–5). In-hospital mortality was higher among sepsis patients at 8.6%, compared to 2.3% for the entire hospital population. The proportion of sepsis patients admitted to the ICU or high dependency units were 10.8%, with higher a rate among HO sepsis (13.2%) than CO sepsis (10.4%). The training set comprised 56,302 (67.9%) admissions with 5436 (9.7%) sepsis episodes and the validation set comprised 26,550 (32.2%) admissions with 2602 (9.8%) sepsis episodes. Measurements of the machine learning models data variables ranged from median 1 to 6 of each variable per hospital admission and data sparsity for the combined training and validation set are shown in the Supplement Table 1.

**Table 1:** Characteristics of the included episodes.

Charcteristics	Total data set	Training set	Validation set
Hospital admissions, No	82,852	56,302	26,550
Patients, No	55,655	40,119	20,863
Female, No. (%)	29,353 (52.7)	21,202 (52.8)	10,858 (52.0)
Age, med (IQR)	63 (44–74)	63 (45–75)	64 (46–75)
Length of stay (days), med (IQR)	3.9 (2.0–7.5)	3.9 (2.0–7.6)	3.9 (2.0–7.5)
Possible screening time points*			
SepsisFinder	1,187,207	795,274	391,933
NEWS2	911,401	612,762	298,639
Charlson comorbidity index, med (IQR)	0 (0–2)	0 (0–2)	0 (0–2)
Prior surgery (30 days), No. (%)	11,877 (14.3)	8059 (14.3)	3818 (14.4)
Suspected infection, No. (%)	19,663 (23.7)	13,292 (23.6)	6371 (24.0)
Sepsis-3 clinical criteria, No. (%)			
All sepsis events	8038 (9.7)	5436 (9.7)	2602 (9.8)
Community-onset sepsis events	6889 (8.3)	4680 (8.3)	2209 (8.3)
Hospital-onset sepsis events	1149 (1.4)	756 (1.3)	393 (1.5)
ICU admission, No. (%)	3853 (4.6)	2605 (4.6)	1248 (4.7)
ICU days, med (IQR)	1.3 (0.9–3.8)	1.2 (0.9–3.6)	1.4 (1.0–4.1)
Bloodstream infection, No. (%)	2613 (3.2)	1715 (3.0)	898 (3.4)
In-hospital mortality, No. (%)	1887 (2.3)	1292 (2.3)	595 (2.2)

*Screenings were only considered possible for time points where there were new measurements which were used in scoring. Median (med), Interquartile Range (IQR), Numbers (No.), National Early Warning Score 2 (NEWS2) and Intensive Care Unit (ICU).

### Algorithm assessment and comparison to other scores

**Figure 1:**
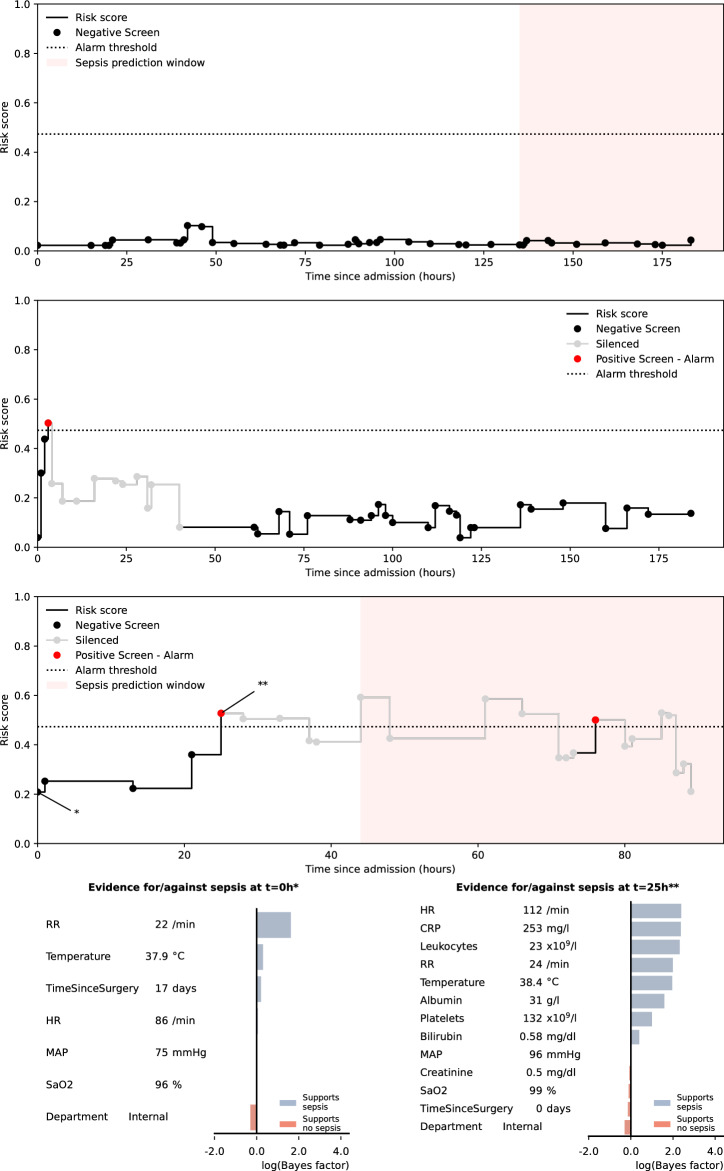
The concept of the sepsis prediction algorithm. The black line represents the SepsisFinder model with predictions marked by black dots. The red dot represents positive alarms. The grey line and dots illustrate silencing 48 h after positive alarms. The alarm threshold is illustrated by the dotted line. The red shaded area represents the time window for considering true positive alarms or false negative predictions. All predictions crossing the alarm threshold outside of the red shaded area were considered false positives. Predictions occurring below the alarm threshold and outside of the red shaded area represent true negative predictions. The upper panel represents a hospital episode with sepsis, but without any positive predictions and only a false negative prediction in the red shaded area. The middle panel shows an episode without sepsis, but with one false positive prediction and several true negative predictions. The lower panel represents a hospital episode with sepsis where both one false positive alarm and one true positive alarm were registered, with model explanations shown below the risk score trace at two selected points: the lowest score in the episode and the first alarm. The explanation plots show the Bayes Factor contributions of the evidence available at the respective times. In the model explanation plots, the blue bars show the degree to which a measurement increases the risk score, while the red bars show the degree to which the risk score is reduced. The Bayes Factor is defined as the ratio of the posterior and prior odds ratios e.g. $B=\frac{P(x|{\varepsilon }{\prime})/P(y|{\varepsilon }{\prime})}{P(x)/P(y)}$ where $x$ is the hypothesis (e.g. sepsis), $y$ is the alternative hypothesis (e.g. no sepsis) and ${\varepsilon }{\prime}$ is the evidence, or a subset thereof. Respiratory rate (RR), heart rate (HR), mean arterial pressure (MAP), c-reactive protein (CRP).

**Figure 2:**
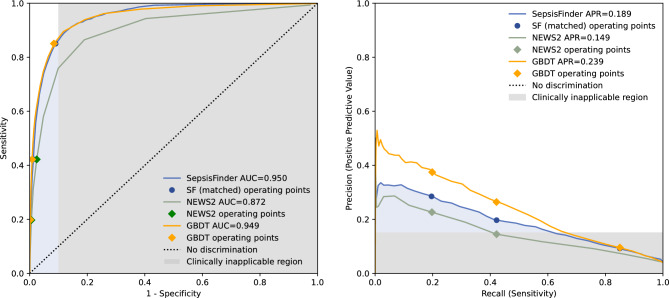
The discriminative performance of the algorithms in the validation set. The left panel shows a receiver operating characteristic curve, and the right panel shows a precision recall curve for the prediction of sepsis within 48 h using SepsisFinder (blue line), the NEWS2 (green line) and the GBDT model (yellow line). Operating alarm thresholds corresponding to NEWS2 equal to 5 and 7 points have been marked for both scores. For SepsisFinder and GBDT, an additional alarm threshold corresponding to approximately 85% sensitivity has been marked. The blue shaded area illustrates the suggested clinically applicable region, and the grey shaded area illustrates the suggested clinically inapplicable region (specificity < 90% and precision < 15%) of model performance. SepsisFinder (SF), Area Under Receiver Operating Characteristic curve (AUC), Area Under Precision Recall curve (APR), National Early Warning Score 2 (NEWS2), and gradient-boosting decision tree (GBDT).

**Figure 3:**
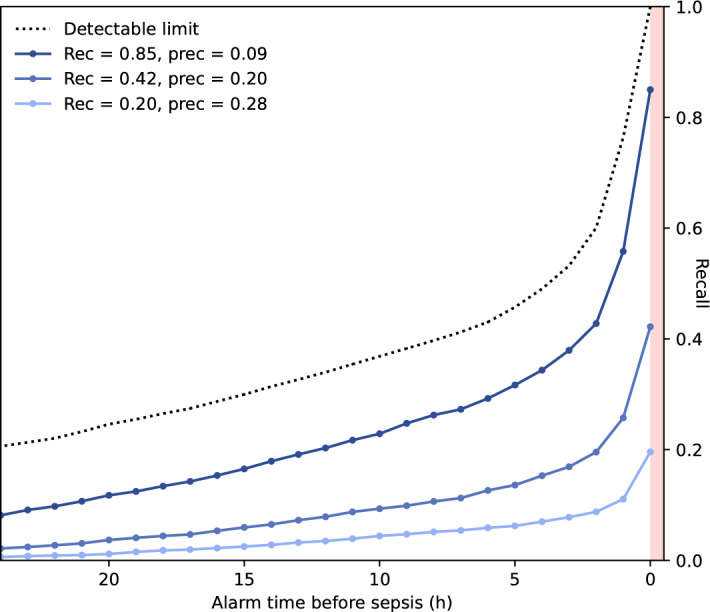
Performance of SepsisFinder in episodes where a sepsis event occurred based on fixed time points 24 h before sepsis onset for three operationalized alarm thresholds. The alarm thresholds were chosen based on sensitivity (recall) matched to NEWS2 equal to 5 points (sensitivity 20%) and 7 points (sensitivity 42%) as well as sensitivity 85%. Since sepsis occurred at all times from admission to discharge, and predictions were only based on data from the current hospital episode, a dotted line has been added to represents the detectable limit for sepsis onset. National Early Warning Score 2 (NEWS2), Recall (Rec), Hours (h), Precision/positive predictive value (Prec).

Depending on the chosen alarm threshold, the machine learned causal probabilistic network (CPN) model –SepsisFinder—produced a median of 5 to 8 screens and mean 0.1 to 0.9 alarms per hospital admission in the validation set (Fig. 1 and Table 2). SepsisFinder predicted sepsis onset within 48 h with excellent discrimination of AUROC 0.950 (95% confidence interval [CI], 0.946–0.954). Due to the highly imbalanced data, area under the precision-recall curve (APR) was 0.189 (95% CI, 0.173–0.201) when assessing the entire population in the validation set (Fig. 2). A machine learning comparator based on a gradient-boosting decision tree (GBDT) model had a similar AUROC of 0.949 (95% CI, 0.945–0.954) with higher APR of 0.239 (95% CI, 0.223–0.254). NEWS2 had an AUROC of 0.872 (95% CI, 0.858–0.877) and an APR of 0.149 (95% CI, 0.138–0.161). If using the combination of organ dysfunction onset and suspected infection criteria as outcome definition, the performance increase slightly for both SepsisFinder (AUROC 0.957 [95% CI, 0.954–0.961]; APR 0.206 [95% CI, 0.191–0.219]), GBDT (AUROC 0.963 [95% CI, 0.959–0.966]; APR 0.294 [95% CI 0.276–0.314]) and NEWS2 (AUROC 0.905 [95% CI, 0.899–0.911]; APR 0.165 [95% CI, 0.153–0.179]) (Supplement Fig. 1). The number of false alarms per true alarm ranged between 2.5 to 9.9 for SepsisFinder, 1.7 to 9.4 for GBDT and 3.4 to 5.9 for NEWS2 depending on the alarm threshold. In episodes where a sepsis event occurred, the fraction of true alarms was plotted as a function of time before sepsis onset (Fig. 3). We observed that the fraction of events that triggered early remained constant during the detectible time limit prior to sepsis onset.

**Table 2:** Screening frequency and predictive performance in the validation set.

Variable	Sepsisfinder			GBDT			NEWS2	
Alarm threshold	Match NEWS2 = 5^a^	Match NEWS2 = 7^b^	Closest to 85% sensitivity	Match NEWS2 = 5^a^	Match NEWS2 = 7^b^	Closest to 85% sensitivity	NEWS2 = 5	NEWS2 = 7
Screens, No	354,583	379,957	244,741	364,836	383,868	253,087	259,504	287,685
Screens per episode, mean; median [IQR]	13.4; 8.0 [3.0–16.0]	14.3; 8.0 [3.0–17.0]	9.2; 5.0 [2.0–11.0]	13.7; 8.0 [3.0–17.0]	14.5; 9.0 [3.0–18.0]	9.5; 6.0 [2.0–12.0]	9.8; 6.0 [2.0–12.0]	10.8; 6.0 [2.0–13.0]
Alarms, No	5576	1798	24,006	4168	1371	22,983	7382	2209
Alarms per episode, mean; median [IQR]	0.2; 0.0 [0.0–0.0]	0.1; 0.0 [0.0–0.0]	0.9; 0.0 [0.0–1.0]	0.2; 0.0 [0.0–0.0]	0.1; 0.0 [0.0–0.0]	0.9; 0.0 [0.0–1.0]	0.3; 0.0 [0.0–0.0]	0.1; 0.0 [0.0–0.0]
False alarms, No	4478	1284	21,795	3070	859	20,772	6313	1709
False alarms per episode, mean; median [IQR]	0.2; 0.0 [0.0–0.0]	0.0; 0.0 [0.0–0.0]	0.8; 0.0 [0.0–1.0]	0.1; 0.0 [0.0–0.0]	0.0; 0.0 [0.0–0.0]	0.8; 0.0 [0.0–1.0]	0.2; 0.0 [0.0–0.0]	0.1; 0.0 [0.0–0.0]
False alarm rate (false alarm/true alarm)	4.1	2.5	9.9	2.8	1.7	9.4	5.9	3.4
Sensitivity	0.422	0.198	0.850	0.422	0.197	0.850	0.422	0.197
Specificity	0.987	0.997	0.910	0.992	0.998	0.917	0.975	0.994
Positive predictive value	0.197	0.286	0.092	0.263	0.373	0.096	0.145	0.226
Negative predictive value	0.996	0.994	0.998	0.996	0.995	0.998	0.994	0.993
Timeliness (all sepsis), mean; median [IQR]^c^	5.6; 1.0 [0.0–8.0]*	5.1; 1.0 [0.0–8.0]†	7.3; 2.0 [0.0–11.0]^	4.4; 0.0 [0.0–5.0]*	2.7; 0.0 [0.0–2.0]†	6.5; 1.0 [0.0–10.0]^	4.3; 0.0 [0.0–4.0]	2.9; 0.0 [0.0–2.0]
Timeliness (HO-sepsis), mean; median [IQR]^c^	17.5; 15.0 [4.0–31.0]**	15.0; 11.0 [5.0–23.5]††	19.8; 18.0 [5.0–33.0]^^	13.3; 7.0 [0.0–24.0]**	7.0; 0.0 [0.0–9.7]††	17.0; 14.0 [1.0–27.5]^^	11.1; 2.0 [0.0–23.0]	9.3; 0.0 [0.0–16.5]

^a^Threshold chosen to match sensitivity obtained for NEWS2 = 5.

^b^Threshold chosen to match sensitivity obtained for NEWS2 = 7.

^c^Timeliness was defined as the time in hours between the true positive alert and sepsis onset in the subset of true positive sepsis cases.

*SepsisFinder compared to NEWS2 = 5, *p* < 0.0001, and GBDT compared to NEWS2 = 5, *p* = 0.74.

**SepsisFinder compared to NEWS2 = 5, *p* < 0.0001, and GBDT compared to NEWS2 = 5, *p* = 0.04.

^†^SepsisFinder compared to NEW2S = 7, *p* < 0.0001, and GBDT compared to NEWS2 = 7, *p* = 0.06.

^††^SepsisFinder compared to NEWS2 = 7, *p* = 0.002, and GBDT compared to NEWS2 = 7, *p* = 0.51.

^SepsisFinder compared to GBDT, *p* = 0.0004.

^^SepsisFinder compared to GBDT, *p* = 0.012.

Gradient-boosting decision tree (GBDT), National Early Warning Score 2 (NEWS2), Numbers (No.), Interquartile Range (IQR) and Hospital-Onset (HO).

With a sensitivity threshold close to 85%, the SepsisFinder predicted sepsis mean 7.3 h and median 2 h (IQR 0-11 h) prior to onset (Table 2). Using the standard cutoff of NEWS2 = 5, sepsis cases were identified mean 4.3 h and median 0 h (IQR 0-4 h) prior to sepsis onset. At a matching sensitivity, the SepsisFinder identified sepsis cases earlier at mean 5.5 h and median 1 h (IQR 0-8 h prior, *p* < 0.0001 for difference compared to NEWS2) prior to sepsis onset. At the same matching sensitivity, GBDT did not identify sepsis cases earlier with mean 4.4 h and median 0 h (IQR 0-5 h prior, *p* = 0.74 compared to NEWS2). With a sensitivity threshold close to 85%, the GBDT predicted sepsis mean 6.5 h and median 1 h (IQR 0–10 h) prior to onset, which was later than SepsisFinder at a matching sensitivity (*p* = 0.0004). These analyses were repeated using a sepsis outcome when both organ dysfunction and suspected infection criteria were fulfilled, which showed a slightly earlier time to alarm for both SepsisFinder, GBDT and NEWS2, as well as less differences in comparison between SepsisFinder and NEWS2 (Supplement Table 2). For this analysis, NEWS2 identified sepsis cases earlier than GBDT (*p* < 0.0001) except for in the subset of HO sepsis patients (*p* = 0.21 for NEWS2 = 5 matching, *p* = 0.94 for NEWS2 = 7 matching). The distributions of the timeliness of alarms before sepsis onset for both SepsisFinder, GBDT and NEWS2 are illustrated in Supplement Fig. 2.

Timeliness of alarm before antibiotic administration in sepsis patients were compared for different alarm thresholds. With a sensitivity threshold close to 85%, the SepsisFinder triggered mean 16.0 h and median 5.5 h (IQR 1.9–22.8 h) vs. GBDT which triggered mean 15.2 h and median 5.1 h (IQR 1.5–21.5 h) prior to antibiotic administration (GBDT vs. SepsisFinder *p* = 0.052). Using an alarm threshold to match the lower sensitivity obtained for NEWS2 = 5 and NEWS2 = 7, the SepsisFinder triggered mean 10.3 h and median 3.2 h (IQR 1.0–11.9 h) and mean 8.2 h and median 2.2 h (IQR 0.3–9.8 h) prior to antibiotic administration, respectively vs. GBDT which triggered mean 9.1 h and median 2.5 h (IQR 0.3–9.0 h) (GBDT vs. SespsisFinder *p* = 0.003) and mean 7.1 h and median 1.5 h (IQR -0.4–7.7 h) (GBDT vs. SespsisFinder *p* = 0.00004) prior to antibiotic administration, for the two matching cutoffs, respectively. For SespsisFinder, this was no different compared to NEWS2. However, GBDT triggered later than NEWS2 (*p* < 0.0001 for both thresholds).

### Performance in subgroups and sensitivity analysis

The robustness of SepsisFinder was assessed by changing the population screened and the timing of screening (Table 3 and Supplement Table 3). The AUROC remained within a similar range, except for episodes resulting in death where AUROC decreased to 0.872. In contrast, the APR displayed greater variation depending on the different subgroups of patients analyzed. The APR increased in shorter episodes of 2 days (APR 0.595) but decreased substantially when restricting screening to episodes longer than 10 days (APR 0.023), in hospital-onset sepsis (APR 0.021) and with more hospital days screened (APR 0.218 when screening up to 5 days). In episodes with culture positivity, defined as confirmed bloodstream infection (BSI), the APR increased to 0.350 compared to APR 0.164 in episodes without a BSI. Furthermore, the APR increased to 0.231 when screening was restricted to the time-period prior to surgery (including episodes where no surgery was performed) compared to APR 0.126 if screening was confined to the time-period after surgery. Sensitivity analyses assessing algorithm performance based on fixed time points using a shorter alarm silencing windows of 12 h or 24 h, as well as considering only predictions occurring -24 to 0 h or -12 to 0 h relative to sepsis onset as true positive alarms are presented in the Supplement Fig. 3. With alarms silencing for 24 h, SepsisFinder had AUROC 0.940 (95% CI, 0.936–0.944) and APR 0.158 (95% CI, 0.146–0.170]), and NEWS2 had AUROC 0.874 (95% CI, 0.866–0.882) and APR 0.131 (95% CI, 0.120–0.142).

**Table 3:** Stratified analyses of SepsisFinder performance in the validation set.

Variable	Discriminatory performance^a^						
Measurement	Num	AUROC	APR	Sens	Spec	PPV	NPV
Episode length^b^							
0–2 days	12,619	0.964	0.595	0.850	0.938	0.442	0.991
2–5 days	7147	0.935	0.034	0.849	0.909	0.029	0.999
5–10 days	4092	0.927	0.018	0.850	0.859	0.012	1.000
10 + days	2692	0.942	0.023	0.850	0.886	0.010	1.000
Days of screening							
1 day	26,550	0.933	0.278	0.850	0.873	0.173	0.995
2 days	26,550	0.950	0.262	0.850	0.895	0.160	0.996
3 days	26,550	0.947	0.235	0.850	0.903	0.135	0.997
4 days	26,550	0.950	0.227	0.850	0.906	0.128	0.997
5 days	26,550	0.948	0.218	0.850	0.907	0.18	0.998
Departments^c^							
Internal	13,857	0.956	0.227	0.850	0.918	0.114	0.998
Surgery	9803	0.936	0.118	0.850	0.895	0.050	0.999
Immune-compromised	2890	0.932	0.170	0.849	0.871	0.099	0.997
Prior to surgery	12,691	0.951	0.231	0.850	0.912	0.135	0.997
Post-surgery	20,150	0.942	0.126	0.850	0.896	0.065	0.999
Bloodstream infection	898	0.937	0.350	0.849	0.889	0.274	0.992
No bloodstream infection	25,652	0.947	0.164	0.850	0.900	0.074	0.998
Patients who died	595	0.872	0.163	0.848	0.754	0.134	0.991
Patients who survived	25,955	0.951	0.192	0.850	0.910	0.088	0.998
Community-onset sepsis^d^	26,157	0.953	0.184	0.850	0.917	0.085	0.999
Hospital-onset sepsis^e^	24,341	0.935	0.021	0.850	0.885	0.013	1.000

^a^Sensitivity, specificity, PPV and NPV are calculated based on the threshold closest to 85% sensitivity.

^b^Days until sepsis, discharge, intensive care unit admission, or death.

^c^Initial admitting department.

^d^Defined as sepsis onset within 4 days of hospital admission. The hospital-onset sepsis episodes are omitted for this analysis.

^e^Defined as sepsis onset after 4 days of hospital admission. The community-onset sepsis episodes are omitted for this analysis. Area Under Receiver Operating Characteristic curve (AUROC), Area Under Precision Recall curve (APR), positive predictive value (PPV), negative predictive value (NPV) and numbers (Num).

## Discussion

This observational study of patients in the non-ICU setting demonstrates that a machine learned CPN model can predict sepsis within 48 h using sparse routine EHR data. The SepsisFinder had good discriminative ability and surpassed the score currently used for sepsis prediction – NEWS2 – in terms of AUROC and APR. In addition, SepsisFinder predicted sepsis onset significantly earlier than both the current practice comparator NEWS2 and a machine learning comparator GBDT for all tested alarm thresholds and triggered up to 5.5 h prior to antibiotic administration indicating opportunities for improving patient care. Since the prevalence of sepsis was low, the false-alarm rate surged when assessing SepsisFinder as a clinical screening tool automatically updating predictions when novel data was available (up to 24 times a day) for the entire hospital admission. In subgroup analyses, the precision improved for shorter hospital episodes, if screening was restricted to the earlier period of the admission and in sepsis with BSI, indicating superior clinical applicability of the score early during hospitalization and in culture positive sepsis.

Implementing a computerized sepsis alert system in clinical practice has been difficult and simple rule-based algorithms have often underperformed^15,16^. Today most clinical decision rules are based on heuristic scoring systems and typically include only a few parameters summarized into a single composite score adapted for manual use. Development of automated machine learned scores based on larger amounts of data, and calibrated to the local situation, have the potential to improve sepsis screening in hospitalized patients^17,18^. This could in turn accelerate a shift from simply detecting when sepsis is present to predicting patients at higher risk of developing sepsis before it occurs. Identifying a high-risk population using dynamic patient factors enables tailored interventions such as increased surveillance, care bundles and earlier treatment, which have been shown to improve patient outcomes^3,4^. In addition, stratification based on risk of sepsis could be used for selecting patients where more advanced or costly testing is warranted, and in which patients it is not, in line with the principles of personalized medicine.

A limitation of several previous studies of machine learning based sepsis prediction tools is the use of administrative data to classify sepsis cases^19–21^. In this study, we used an objective sepsis classification based on clinical data, which is more reliable, less prone to bias, and robust over time^22,23^. Our classification method was previously developed in the same research database as this study, meaning the sepsis outcome used as basis for the predictions had been thoroughly validated^24^. The major strength is that this approach captures the entire intended screening population and generates results which are easier to compare and more generalizable to other settings^25^.

We focused on patients in the emergency department or non-ICU wards. The demands of sepsis screening tools differ depending on the screening population, both with regards to data availability (high-resolution or low-resolution) and screening frequency (single, intermittent, or continuously)^26^. As shown in the most comprehensive systematic review of machine learning sepsis prediction models to date, most published studies have focused on ICU patients^11^. The ICU constitutes a data rich environment where monitoring of physiological parameters is performed continuously, and biomarkers are assessed with regular and close intervals in most patients. This can be exploited in model learning to improve predictions and physiological parameters have been shown to be both temporally and differentially expressed in septic ICU patients^27,28^. Yet only a small proportion of sepsis develop in the ICU and a major clinical benefit lies in identifying patients earlier in the disease trajectory before ICU admission^29,30^. As an example, a population-based point-prevalence assessment of the Sepsis-3 criteria in all hospitalized patients receiving intravenous antibiotics in two large regions in Sweden found that only 2.8% of sepsis patients had their antibiotics initiated at the ICU^30^. This suggests that most sepsis cases are already detected, or at least have received the most crucial treatment intervention, before being admitted to the ICU. Furthermore, a large systematic review and meta-analysis found that the pooled incidence of hospital-treated sepsis cases was 189 per 100,000 person-years, while the pooled incidence of ICU-treated sepsis cases was only 58 per 100,000 person-years^29^. In non-ICU wards, data availability is sparse as illustrated by our findings of an overall measurement frequency ranging between 0 to 1.9 per 24 h. During such circumstance continuous screening does not make sense and our score was designed to update on regularly once every hour if new data was available, reflecting the workflow of collecting vital and laboratory parameters in general hospital wards. To avoid alert fatigue and simulate a situation where clinicians are thought to act on the information, we also chose to silence each positive alarm.

Risk models can be described as existing across two spectra: from completely knowledge based to completely data driven, and from simple to calculate (e.g. an additive score) to exceedingly complex (e.g. output of a deep neural network, or the GBDT described in this study). Our primary analysis focused on a score based on a supervised CPN model, which sits somewhere in the middle of each spectrum. CPNs consist of network of nodes, which may represent concepts, measurements, or symptoms, and can be observable or unobservable. The nodes are linked by causal links, which are described mathematically as conditional probability tables. The conditional probability tables describe the a priori beliefs of the network, which confer the inherent ability to handle missing data. When evidence is introduced into one or more nodes, the beliefs throughout the network are updated according to the axioms of probability. It is possible to learn both the structure and the conditional probability tables directly from data, or to manually specify part or all of the model. This allows for the fusion of data and knowledge, implementing constraints or other structural features based on expert knowledge, while fine-tuning the probability tables with empirical data^31^. All machine learning models are subject to the bias-variance tradeoff. One benefit of the ability to constrain the model is the avoidance of overfitting, although this comes potentially at the cost of increased accuracy. As more evidence becomes available, it is also possible to adapt all or part of the model using a penalized learning approach, where weights can be specified for the existing conditional probabilities or sections of the model held invariant, prior to performing learning^32^. In contrast, the GBDT is completely data driven and requires complex calculations to compute. To avoid overfitting, hyperparameters can be adjusted to reduce model complexity and are typically selected in cross-validation via a grid search across hyperparameter space.

Explainability is an important factor to develop trust in clinical decision support systems^33,34^. In addition to their adaptability, although the calculations performed are not trivial, CPNs are interpretable models, which are inherently explainable^33^. Once evidence has been propagated throughout the network, it is possible to read off the probabilities associated with any of the nodes. For example, in addition to the sepsis prediction node used as a predictor in this study, it is possible to read off probabilities describing the sepsis severity, probability of bacteraemia and of 30-day mortality. Similarly, it is possible to determine the impact of any one piece (or combination) of evidence on the probability of a particular state of a particular node as shown in the lower panel of Fig. 1. The principle of the model has similarities with clinical reasoning, making it easy to understand compared to other complex models, which are important aspects when convincing clinicians to trust the predictions^35^. This is in contrast to other machine learning models, such as the GBDT, which are not interpretable. However, significant steps have been taken towards explainability for such models, such as the use of Shapley additive explanation (SHAP) or local interpretable model-agnostic explanation (LIME) methods^36,37^. To our knowledge, only a few studies using CPN models to predict sepsis have been reported, but without any clinically realistic performance evaluation^38,39^.

The SepsisFinder and GBDT models presented contrasting performance, with the GBDT showing higher precision than SepsisFinder, but its triggers were less timely. At sensitivity thresholds above approximately 60% the precision was similar between the two models. The clinical utility of a scoring system is dependent not only on its precision, but also on whether it would allow an earlier intervention to be made. In the SepsisFinder and GBDT results, there appears to be a tradeoff between precision and timeliness. If we hypothesize that for each patient developing sepsis there is a period of deterioration of indeterminate length leading to their classification as septic, it follows that detection earlier in a given period provides more scope for false positives, due to increased difficulty in discriminating these cases from similarly ill, non-septic patients. Since time to treatment is critical in sepsis, we argue from a clinical standpoint that a slightly higher level of false positive screens is acceptable if the alternative is to identify sepsis closer to onset^5^.

There have been prior works of sepsis prediction models for the non-ICU setting, but many of these studies focus on the technical rather than clinical aspects, use outdated sepsis definitions not accounting for chronic organ dysfunction, or limit their evaluation to specific patient populations^19,40–45^. In this study, we put emphasis on simulating the performance as it would be if it was implemented in a real-world setting. We used sepsis related organ dysfunction based on the change in SOFA score as our main outcome to better reflect the pathophysiological onset of sepsis, rather than predicting the time of clinical identification based on cultures or antibiotic administration. In addition, we evaluated the score in the intended screening population, i.e., all patients admitted to the hospital. The AUROC of SepsisFinder was within a similar range, or higher, than reports of sepsis prediction models based on other machine learning techniques^11^. Many studies report a cumulative maximum score, meaning no limit on how early sepsis is detected, which has low clinical applicability since the positive alarm can be unrelated in time to the actual sepsis episode^12,46^. We only considered alarms within 48 h of sepsis onset as true positives, ensuring they were associated with the sepsis event. Equally important for screening in a clinical environment is the proportion of true positive alarms among all positive alarms, but APR curves have not been frequently reported in machine learning models for sepsis^12,20,46,47^. The precision (positive predictive value) is dependent on the prevalence of outcome. In our study, 9.8% of patients in the validation set experienced a sepsis event, which is within the similar range of other studies^12,13,47^. Most sepsis events developed within the first days of admission and only 1.4% of the total cohort contained a hospital-onset sepsis event occurring later during the hospitalization. This partly explains the lower precision, which decreased further with episode length, suggesting better applicability of SepsisFinder early during hospitalization. Although most machine learning models generate a continuous probability score between zero to one, choosing a threshold is usually required to facilitate clinical usage. We compared three operational alarm thresholds, to illustrate the tradeoff between sensitivity, specificity, and precision, and choosing a final threshold depends on the desired purpose of the screening. In most circumstances, since sepsis is a medical emergency associated with substantial mortality, high sensitivity at the expense of precision would likely be preferred.

For individual patient level predictions in clinical practice, factors such as the false alarm rate and alarm fatigue needs to be considered^48^. To better illustrate the applicability of the prediction model in this setting, we used a previously reported method and selected two margins for lowest acceptable clinical performance (specificity < 90% and precision < 15%, respectively) (Fig. 2)^49^. Compared to NEWS2, the SepsisFinder and GBDT performed better on all levels, however, at thresholds with sensitivity approximately > 60%, the precision decreased below the suggested margin. In subgroup analyses of the study population, shorter episode length, screening only for the first few days of hospital admission and community-onset sepsis was associated with higher APR of the SepsisFinder suggesting that the clinical usage may be more relevant in these situations. This illustrates that applying sepsis prediction scores on all hospitalized patients is difficult, and limiting the use of SepsisFinder to subgroups, may hold better promise. In addition, as demonstrated by higher APR, the SepsisFinder performed better in culture positive sepsis and in patients before surgery, which indicates better applicability in patients with “classical” sepsis.

Our study has several limitations. The SepsisFinder was developed using observational data from a single center, and the external generalizability needs to be confirmed. Even though we used a large and representative hospital population, the score is not universal and needs calibration using local data before implementation in another setting, which is a necessity with most predictive machine learning models^50^. On the other hand, we trained and tested our model using a copy of the operational EHR system without major changing of variables, hence, simulating a realistic clinical use-case and facilitating implementation. We also used features of patient data which are generally collected and stored in most EHR systems and present a transparent framework for building and assessing machine learning models aimed at clinical practice, which we encourage others to use. As with all similar machine learning scores, the model performance is dependent on correct and accessible input data and we cannot rule out that missing variables, or differences in documentation of clinical data within the hospital, affected our results. Then again, missing data in EHR systems is generally not missing at random, but reflective of clinical decisions, and studies indicate that methods to reduce missing data in sepsis machine learning prediction models does not improve performance^45^.

In conclusion, a machine learned CPN algorithm (SepsisFinder) trained on sparse routine EHR data predicted sepsis onset within 48 h with better discrimination and earlier than NEWS2 outside the ICU-setting. Compared to a GBDT model, the precision was somewhat lower, but the SepsisFinder triggered earlier which we believe is of clinical relevance. The precision of SepsisFinder increased if screening was restricted to the time directly following admission suggesting that screening may primarily be warranted for this period. Identifying a high-risk population with this method could be used to tailor clinical interventions and improve patient care, but further implementation studies are needed.

## Methods

### Design, data source and study population

This was a cohort study including patients from the Karolinska University Hospital, Sweden. The study was approved by the Regional Ethical Review Board in Stockholm (approval number 2016/22,309–32 and 2012/1838–31/3) and performed in accordance with the permission. According to national standards for similar studies, the Regional Ethical Review Board in Stockholm gave their approval to the study with a waiver of consent from participants. Data were obtained from regularly entered information in the EHR system, stored in a research database named the Health Bank^51^. The longitudinal database structure is a duplicate of the currently in-use operating EHR system and consists of all medical records from anonymized patients that received care at the hospital until the beginning of 2014. All adult patients ≥ 18 years admitted to the hospital for ≥ 24 h between July 2012 and December 2013 were included. Due to data availability, patients were excluded if admitted to an obstetric ward. The cohort was divided into a training set (July 2012–June 2013) and a validation set (July 2013–December 2013). Data on demographics, department, length-of-stay, vital parameters, laboratory parameters, microbiological inquiries, administered antibiotics and in-hospital mortality was collected for each hospital episode. Data on International Classification of Diseases (ICD)-10 codes and surgical procedure codes were retrieved up to 5 years before inclusion.

Sepsis onset was determined according to a previously validated rule-based classification based on the Sepsis-3 criteria. The classification algorithm has previously shown sensitivity 88.7%, specificity 98.5% and positive predictive value 88.1% when using physician review of medical records as gold standard^24^. In accordance with the Sepsis-3 criteria, suspected infection was defined as having any microbiological culture taken and at least 2 doses of antimicrobials administered and increase in Sequential Organ Failure Assessment (SOFA) score by ≥ 2 points compared to a baseline value. Onset of sepsis was defined as the time point when the patient fulfilled the organ dysfunction criteria.

### Machine learning model

For our main analysis, a causal probabilistic network (CPN) model – SepsisFinder – that has previously been used to predict bloodstream infection and 30-day mortality was adapted and re-trained to predict sepsis onset (Supplement Methods 1)^52,53^. Variables included in the model were routine measurements of heart rate, mean arterial pressure, respiratory rate, peripheral oxygen saturation, oxygen delivery (liters/minute), mental status, c-reactive protein, white blood cell count, platelets, bilirubin, creatinine, urea, albumin, lactate, HCO3, pH, current department, and time since surgery. To adapt the model for sequential data, we introduced decay factors which limited the model’s belief in a measurement as time passed since the measurement was recorded. Measurements were filled forward without backfilling missing measurements. Only the most recent measurement, along with the time since it was measured, was used at each screening. As an input for model training, a discretized time-to-sepsis label was used.

In addition to the SepsisFinder model, we trained a gradient-boosting decision tree (GBDT) model using the LightGBM framework as a purely data-driven machine learning comparator^54^. The GBDT model was trained using the same data available to SepsisFinder. Basic hyperparameter tuning was performed via a grid search across the following parameters: max depth, number of iterations, l1 and l2 regularization. The best hyperparameters were selected based on tenfold cross-validation using the training set.

### Performance assessment

The intended use case was a clinical screening tool for assessing the risk of sepsis within the next 48 h. Discrimination was calculated using AUROC and APR based on individual screens, with bootstrapped confidence intervals (CI) (Supplement Methods 2). A prediction was generated on all hospitalizations once every hour, providing a new variable was registered, from admission to either sepsis, ICU-admission, discharge, or death. The performance of SepsisFinder was compared with the GBDT model to contrast a different model (machine learning comparator), and with the routinely used warning score NEWS2 to reflect how sepsis prediction is performed today (current practice comparator)^6^. The NEWS2 was calculated for every time point at which at least one of the score’s components were available. To reflect how NEWS2 is used in practice, missing values were not carried forward from earlier time points. The alarm of both SepsisFinder, GBDT and NEWS2 was silenced for 48 h after each positive trigger, to simulate a situation where healthcare providers are thought to act on a threshold-based warning system (Fig. 1). Three operating points for SepsisFinder and GBDT were chosen to match the sensitivity of the standard clinical decision-making thresholds for NEWS2: NEWS2 = 5 and NEWS2 = 7, and the threshold that gave closest to 85% sensitivity. Timeliness of the true positive alert, defined as hours before sepsis onset, was assessed for each threshold in the true positive cases, and compared using the Mann–Whitney U test. To further evaluate clinical utility of SepsisFinder and GBDT, timeliness of alarm before antibiotic administration in the true positive sepsis cases were also assessed. Two-sided P-values < 0.05 were considered statistically significant. Analyses were performed in R and Python^55^.

### Subgroup and sensitivity analysis

Further analysis was restricted to the SepsisFinder model. The SepsisFinder model was assessed in subgroups in the validation set to evaluate its robustness in different clinical scenarios and identify areas for potential applicability. The following subgroups were considered: (I) episode length of 0–2 days, 2–5 days, 5–10 days and longer than 10 days, (II) sepsis screening for the 1, 2, 3, 4 and 5 days of admission, (III) admission department category, defined as Internal, Surgery or Immunocompromised, (IV) episodes with and without surgery (divided into pre- and post-surgery), (V) episodes with and without significant bloodstream infection (BSI) at any point in the admission^24^, (VI) episodes with survivors and non-survivors, and (VII) sepsis-onset time before or after 4 days of admission defined as community-onset (CO) sepsis or hospital-onset (HO) sepsis, in each case including all other patients not classified as either of these. Prior surgery was defined based on administrative codes and episodes with at least one of these was split on the day of the first surgery. If a patient had surgery immediately before the start of the hospital episode (within 7 days), this was classified as post-surgery risk-time. Sensitivity analyses were also performed to investigate the effect of the window in which alarms are considered true positives (12 h, 24 h compared with base case of 48 h), of the time for which alarms were silenced (12 h, 24 h compared with base case of 48 h), and the effect of the outcome definition (sepsis onset defined as the time point when both organ dysfunction and suspected infection criteria met compared with the base case of organ dysfunction only).

## Supplementary Information

Supplementary Information.

## Data Availability

Data from deidentified electronic medical records are not freely available due to protection of the personal integrity of the participants. Access to patient level data requires a Swedish ethical permit and an agreement with the research organization, Department of Computer and Systems Sciences, Stockholm University, holder of the data. Any requests regarding data for this study can be sent to the corresponding author.
