# Supplementary material for: Predicting sepsis onset using a machine learned causal probabilistic network algorithm based on electronic health records data
Source: Sci Rep. 2023 Jul 20;13:11760. doi: 10.1038/s41598-023-38858-4 (PMC10359402; doi:10.1038/s41598-023-38858-4)
Supplement: Supplementary file 1 — Supplementary Information. [file 41598_2023_38858_MOESM1_ESM.pdf]

Supplement

Predicting sepsis onset using a machine learned causal probabilistic network algorithm based on electronic health records data

**Supplement Methods 1.** The machine learning model	Page 2
**Supplement Methods 2.** Performance assessment	Page 3
**Supplement Table 1.** Data sparsity in the combined training and validation data set	Page 4
**Supplement Table 2.** Screening frequency and predictive performance characteristics in the validation data set for a sepsis outcome where both organ dysfunction and suspected infection criteria met	Page 5
**Supplement Table 3.** Stratified analyses in the validation set for a sepsis onset definition where both organ dysfunction and suspected infection criteria are met (n=26550)	Page 6
**Supplement Figure 1.** Algorithm performance in the validation set for a sepsis outcome where both organ dysfunction and suspected infection criteria met	Page 7
**Supplement Figure 2.** Timeliness of algorithm detection of sepsis compared to NEWS2 in the validation data set	Page 8
**Supplement Figure 3.** Algorithm performance based on fixed time points in episodes where a sepsis event occurred in the validation set	Page 11

Supplement Methods 1. The machine learning model

Data preparation

The electronic health record (EHR) data was prepared by mapping the EHR variables to those used by the model, and merging where model variables could be described by more than one EHR variable. For example, the mental status variable in the causal probabilistic network (CPN) model uses information that could be encoded as the Glasgow Coma Scale (GCS) or as alert/not alert as a component of a clinical score such as NEWS. Where variables were recorded with multiple encodings at a single time point, the most accurate/highest resolution source was used, e.g. GCS took preference over alert/not alert. Individual departments were mapped into the following categories: Internal (Internal medicine, Geriatrics, Urology), Surgery (General surgery, Neurosurgery, Orthopaedic surgery, Thoracic surgery) or Immunocompromised (Oncology, Haematology, Transplant). Data were resampled and aggregated into one-hour time windows. Where multiple measurements were recorded for a single variable during the window, the most recent was kept. As an input for model training, a discretized time-to-sepsis label was used. We were interested in predictions of deterioration within 48h of the event, and particularly within 24h so we labelled time points as sepsis occurring within 48h, 24h, 20h, 16h, 12h, 8h, 4h. Due to the different frequencies with which the included variables were recorded, new features were added to encode the time since the last measurement of each type, for a given care episode, in hours. This enabled the belief in a measurement to be adjusted according to its recency. Data for each hospital episode were truncated at sepsis onset, ICU admission, discharge, or death.

Model adjustments and training

The original CPN model was designed to use a snapshot of patient data available at a specific time in their course of illness – the point at which cultures are drawn. The original training data set consisted of patients with suspected community-acquired infection. For this study, the model was adjusted and the input variables included were: heart rate, mean arterial pressure, respiratory rate, peripheral oxygen saturation, oxygen delivery (liters/minute), mental status, c-reactive protein, white blood cell count, platelets, bilirubin, creatinine, urea, albumin, lactate, HCO3, pH, current department and time since surgery. To adapt the model for sequential data, we introduced time-dependence in the form of decay factors which limited the model's belief in a measurement as time passed since the measurement was recorded. Measurements were filled forward (forward imputation) without backfilling missing measurements. Only the most recent measurement, along with the time since it was measured, was used at each time point. A new binary outcome variable, SepsisPrediction, was also introduced as a child of the Sepsis and Systemic Inflammatory Response Syndrome (SIRS) nodes. Features for the current department (Internal, Surgery, Immunocompromised, Other) and for the time since most recent surgical intervention were also added as parents to the SepsisPrediction outcome, to allow for different severity patterns according to department, and the potential confounding effect of recent surgery. The model was trained in Hugin version 8.8 (Hugin Expert, Aalborg, Denmark) using the inbuilt expectation-maximization algorithm. Hugin allows individual nodes or individual states of a node to be held invariant during learning. Learning was performed in the SepsisPrediction node, with the remainder of the model considered invariant.

Supplement Methods 2. Performance assessment

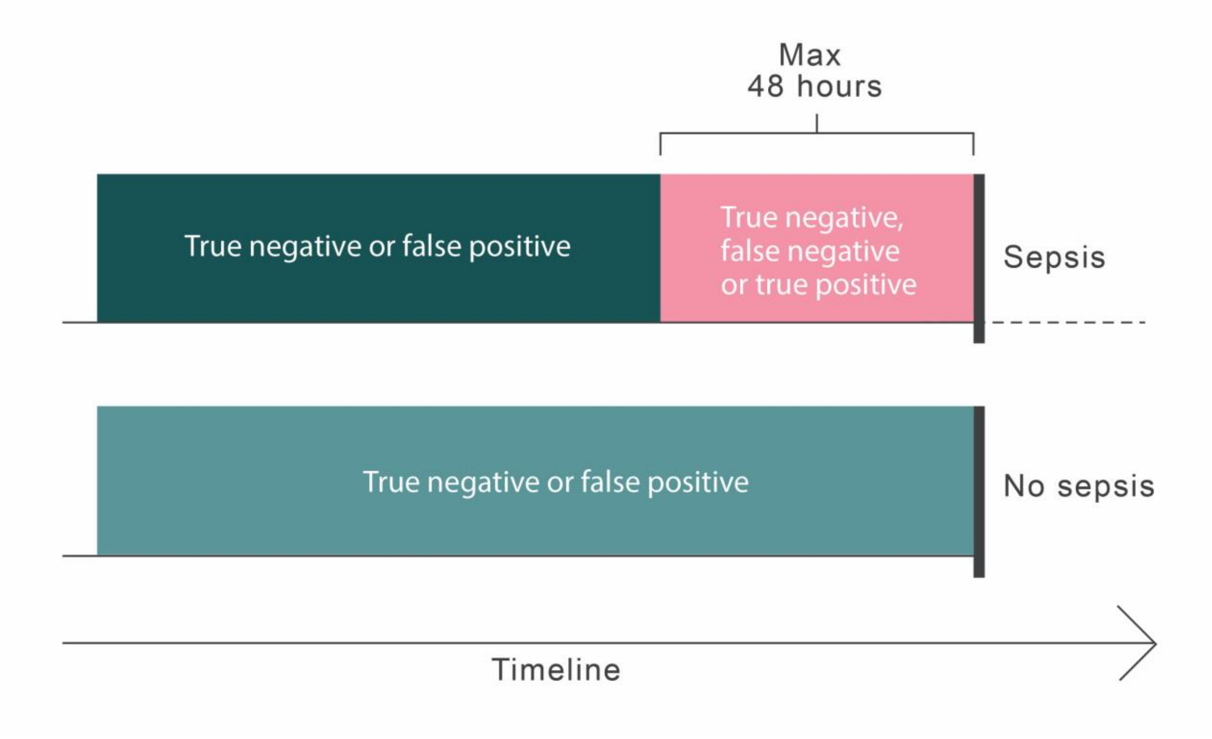

Legend: To construct the AUROC and APR, the elements of the 2x2 contingency table were defined based on each screen. A true positive screen was where the score was above the threshold, and within 48h of sepsis onset. A single episode could have a maximum of one true positive. A false positive screen was where the score was above the threshold for any screening not within 48h of sepsis onset. A single episode could have multiple false positives. A true negative screen was where the score was below the threshold and was not the final screen in a sepsis episode. A single episode could have multiple true negatives. A false negative screen was where the score was below the threshold and was the final screen in a sepsis episode. A single episode could have a maximum of one false negative. The 2.5th and 97.5th percentile confidence intervals for AUROC and APR were calculated using 1000 bootstrap resamples of the data. Resampling was done on a per-episode basis.

Abbreviations: Area Under Receiver Operating Characteristics curve (AUROC) and Area Under Precision Recall curve (APR).

Supplement Table 1. Data sparsity in the combined training and validation data set

Parameter	Measurements (total), No.	Measurements per episode, median [IQR]	Overall measurement frequency per 24h	Measurement frequency per episode*, median [IQR]
**MAP**	671939	6.0 [2.0 - 12.0]	1.9	2.4 [1.5 - 3.8]
**Heart rate**	662874	6.0 [2.0 - 12.0]	1.9	2.4 [1.5 - 3.6]
**Temperature**	658701	5.0 [2.0 - 11.0]	1.9	2.3 [1.6 - 3.4]
**SpO2**	576959	5.0 [2.0 - 10.0]	1.6	2.2 [1.3 - 3.4]
**Respiratory rate**	137606	2.0 [1.0 - 4.0]	0.4	0.8 [0.3 - 2.1]
**Mental Status**	9885	3.0 [1.0 - 5.0]	0.03	1.0 [0.4 - 1.8]
**Creatinine**	188842	2.0 [1.0 - 3.0]	0.5	0.7 [0.4 - 1.2]
**Leukocytes**	184852	2.0 [1.0 - 3.0]	0.5	0.7 [0.4 - 1.2]
**Neutrophil fraction**	23261	1.0 [1.0 - 2.0]	0.07	0.4 [0.2 - 0.8]
**CRP**	161318	2.0 [1.0 - 4.0]	0.5	0.7 [0.4 - 1.1]
**Platelets**	155794	1.0 [1.0 - 3.0]	0.4	0.6 [0.4 - 1.1]
**Albumin**	94764	1.0 [1.0 - 2.0]	0.3	0.5 [0.3 - 1.0]
**Urea**	17676	1.0 [1.0 - 2.0]	0.05	0.3 [0.2 - 0.6]
**Lactate**	170	1.0 [1.0 - 2.0]	0	0.14 [0.09 - 0.29]

*Only for episodes with at least one measurement of the type.

Abbreviations: Peripheral oxygen saturation (SpO2), Numbers (No.) and Interquartile Range (IQR).

Supplement Table 2. Screening frequency and predictive performance characteristics in the validation set for a sepsis onset definition where both organ dysfunction and suspected infection criteria are met

Variable	SepsisFinder			GBDT			NEWS2	
**Alarm threshold**	**Match NEWS=5[a]**	**Match NEWS=7[b]**	**Closest to 85% sensitivity**	**Match NEWS=5[a]**	**Match NEWS=7[b]**	**Closest to 85% sensitivity**	**NEWS2=5**	**NEWS2=7**
**No. screens**	356917	382188	263654	371860	387531	279829	260808	289559
**Screens per episode, mean; median [IQR]**	13.4; 8 [3-16]	14.4; 9 [4-17]	9.9; 6 [2-12]	14.0; 8 [3-17]	14.6; 9 [4-18]	10.5; 6 [3-13]	9.8; 6 [2-12]	10.9; 6 [2-13]
**No. alarms**	5698	1938	20927	3710	1347	18423	7507	2276
**Alarms per episode, mean; median [IQR]**	0.2; 0 [0-0]	0.1; 0 [0-0]	0.8; 0 [0-1]	0.1; 0 [0-0]	0.1, 0 [0-0]	0.7, 0 [0-1]	0.3; 0 [0-0]	0.1; 0 [0-0]
**No. false alarms**	4506	1371	18715	2519	780	16210	6340	1720
**False alarms per episode, mean; median [IQR]**	0.2; 0 [0-0]	0.1; 0 [0-0]	0.7; 0 [0-1]	0.1; 0 [0-0]	0.0, 0 [0-0]	0.6, 0 [0-1]	0.2; 0 [0-0]	0.1; 0 [0-0]
**False alarm rate (false alarm/true alarm)**	3.8	2.4	8.5	2.1	1.4	7.3	5.4	3.1
**Sensitivity**	0.458	0.218	0.850	0.458	0.218	0.850	0.458	0.218
**Specificity**	0.987	0.996	0.928	0.993	0.998	0.942	0.975	0.994
**Positive predictive value**	0.209	0.293	0.106	0.321	0.421	0.120	0.155	0.244
**Negative predictive value**	0.996	0.995	0.998	0.996	0.995	0.999	0.995	0.993
**Timeliness (All sepsis), mean; median [IQR][c]**	6.5; 2 [0-9]*	5.8; 2 [0-9]†	8.5; 3 [1-13]	4.6; 1 [0-5]‡	3.3; 0 [0-2]‡	7.7; 2 [1-12]	6.4; 2 [0-8]	4.7; 1 [0-4]
**Timeliness (HO-sepsis), mean; median [IQR][c]**	16.3; 12 [2-26]**	15.0; 11 [4-24]††	19.1; 17 [5-32]	11.3; 6 [0-17]‡‡	10.2; 2.5 [0-16]‡‡‡	17.7; 15 [3-30]	14.4; 9 [0-27]	10.7; 3 [0-19]

[a]Threshold chosen to match sensitivity obtained for NEWS2=5.
[b]Threshold chosen to match sensitivity obtained for NEWS2=7.
[c]Timeliness was defined as the time in hours between the true positive alert and sepsis onset in the subset of true positive sepsis cases.
*Compared to NEWS2 = 5, p=0.04.
**Compared to NEWS2 = 5, p=0.07.
†Compared to NEWS2 = 7, p=0.0003.
††Compared to NEWS2 = 7, p=0.02.
‡Compared to NEWS2 = 5 or 7, as appropriate, p<0.0001
‡‡Compared to NEWS2 = 5, p=0.21
‡‡‡Compared to NEWS2 = 7, p=0.94

Abbreviations: Gradient-boosting Decision Tree (GBDT), National Early Warning Score (NEWS2), Numbers (No.), Interquartile Range (IQR) and Hospital-Onset (HO).

Supplement Table 3. Stratified analyses in the validation set for a sepsis onset definition where both organ dysfunction and suspected infection criteria are met (n=26550)

**Variable**	**Discriminatory performance[a,f]**						
**Measurement**	Num.	AUROC	APR	Sens	Spec	PPV	NPV
**Episode length[b]**							
0-2 days	12572	0.971	0.620	0.850	0.949	0.482	0.992
2-5 days	7178	0.933	0.046	0.848	0.924	0.038	0.999
5-10 days	4105	0.932	0.021	0.850	0.880	0.014	1.000
10+ days	2695	0.952	0.023	0.845	0.919	0.012	1.000
**Days of screening**							
1 day	26550	0.944	0.294	0.850	0.896	0.190	0.995
2 days	26550	0.957	0.278	0.850	0.916	0.179	0.997
3 days	26550	0.955	0.253	0.850	0.820	0.150	0.997
4 days	26550	0.958	0.244	0.850	0.923	0.142	0.998
5 days	26550	0.955	0.235	0.850	0.924	0.133	0.998
**Departments[c]**							
Internal	13857	0.962	0.242	0.850	0.934	0.129	0.998
Surgery	9803	0.945	0.130	0.850	0.916	0.058	0.999
Immune-compromised	2890	0.951	0.190	0.849	0.919	0.127	0.998
**Prior to surgery**	12691	0.957	0.242	0.850	0.927	0.150	0.998
**Post-surgery**	20150	0.951	0.147	0.850	0.917	0.074	0.999
**Bloodstream infection**	898	0.951	0.362	0.849	0.923	0.315	0.993
**No bloodstream infection**	25652	0.954	0.180	0.850	0.924	0.087	0.999
**Patients who died**	595	0.899	0.176	0.849	0.794	0.140	0.992
**Patients who survived**	25955	0.958	0.208	0.850	0.930	0.102	0.998
**Community-onset sepsis[d]**	26110	0.962	0.200	0.850	0.935	0.098	0.999
**Hospital-onset sepsis[e]**	24388	0.938	0.025	0.850	0.903	0.016	1.000

[a]Please note that changing the sepsis onset definition leads to differences in classification of episode lengths before sepsis onset, as well as differentiation between community and hospital-onset sepsis.
[b]Days until sepsis, discharge, intensive care unit admission, or death.
[c]Initial admitting department.
[d]Defined as sepsis onset within 4 days of hospital admission. The hospital-onset sepsis episodes are omitted for this analysis.
[e]Defined as sepsis onset after 4 days of hospital admission. The community-onset sepsis episodes are omitted for this analysis.
[f]Sensitivity, specificity, PPV and NPV are calculated based on the threshold closest to 85% sensitivity.

Abbreviations: Area Under Receiver Operating Characteristic curve (AUROC), Area Under Precision Recall curve (APR), positive predictive value (PPV), negative predictive value (NPV) and numbers (Num).

Supplement Figure 1. Algorithm performance in the validation data set for a sepsis outcome where both organ dysfunction and suspected infection criteria met

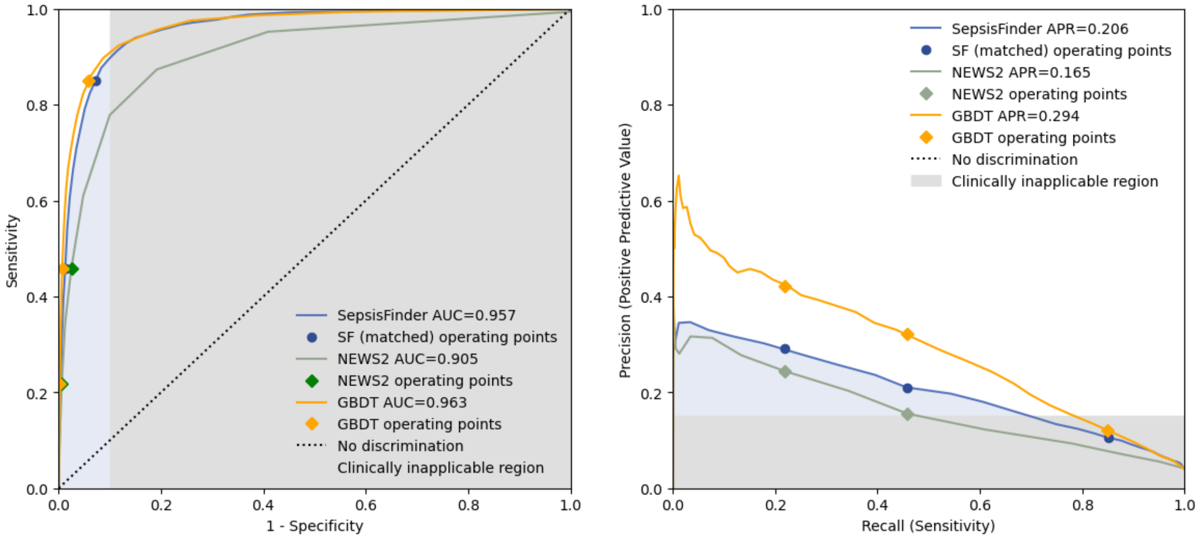

Legend: The left panel shows a receiver operating characteristic curve, and the right panel shows a precision recall curve, for the prediction of sepsis within 48 hours using SepsisFinder (blue line), the NEWS2 (green line) and the GBDT model (yellow line). Operating alarm thresholds corresponding to NEWS2 equal to 5 and 7 points have been marked for both scores. For SepsisFinder and GBDT, an additional alarm threshold corresponding to approximately 85% sensitivity has been marked. The blue shaded area illustrates the suggested clinically applicable region, and the grey shaded area illustrates the suggested clinically inapplicable region (specificity <90% and positive predictive value <15%) of model performance. Sepsis Finder had AUROC 0.957 (95% CI, 0.954-0.961) and APR 0.206 (95% CI, 0.191-0.219). NEWS2 had AUROC 0.905 (95% CI, 0.899-0.911) and APR 0.165 (95% CI, 0.153-0.179). GBDT had AUROC 0.963 (95% CI, 0.959-0.966) and APR 0.294 (95% CI, 0.276-0.314).

Abbreviations: SepsisFinder (SF), Gradient-boosting Decision Tree (GBDT), Area Under Receiver Operating Characteristic curve (AUC), Area Under Precision Recall curve (APR) and National Early Warning Score 2 (NEWS2).

Supplement Figure 2. Timeliness of algorithm detection of sepsis compared to NEWS2 in the validation data set

A: All sepsis episodes

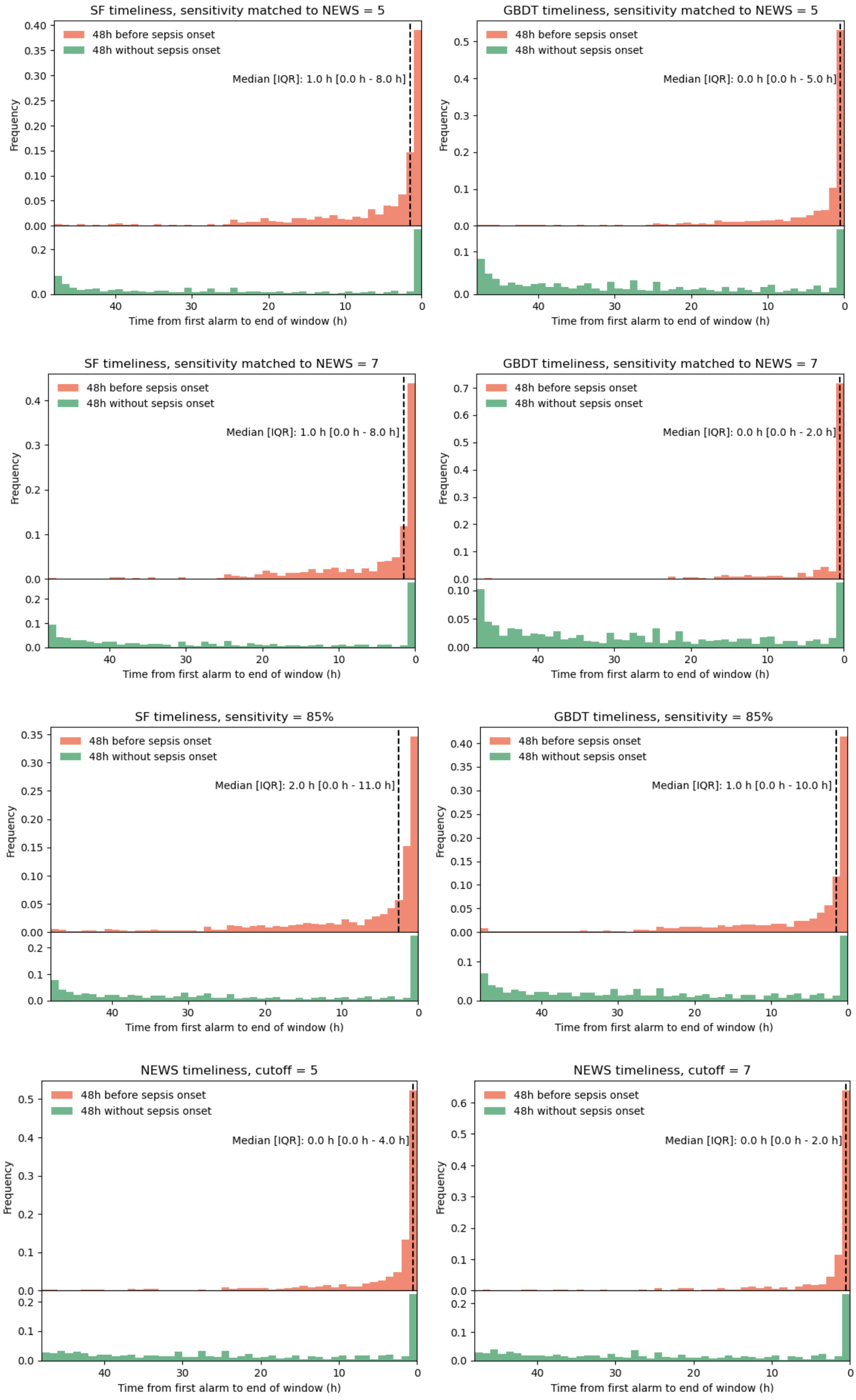

B: Only hospital-onset sepsis episodes

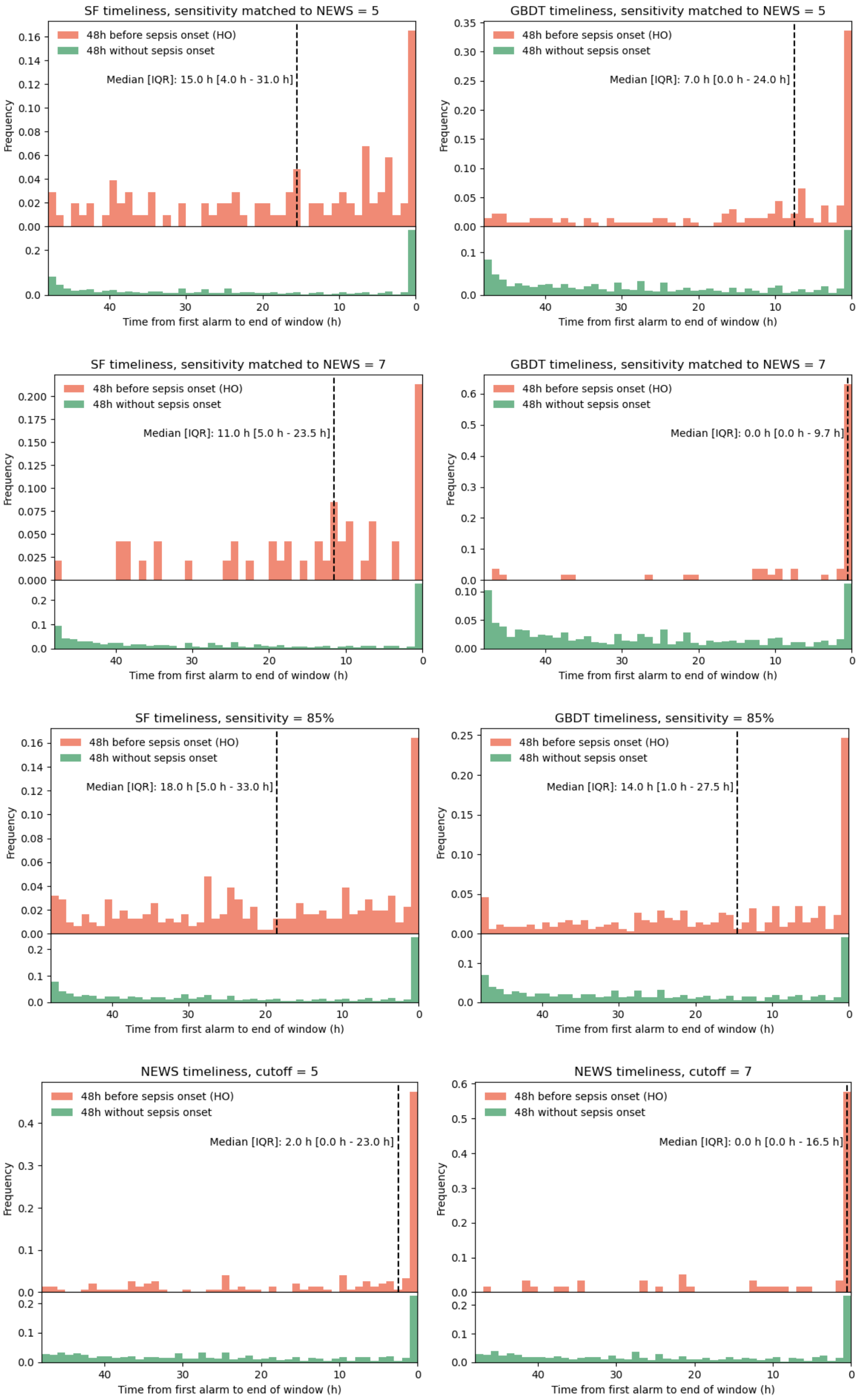

Legend: The orange bars represent the distribution of timing of first alarm (SepsisFinder, GBDT and NEWS2) in the 48 hours before a sepsis event. The green bars represent the distribution of false alarms in 48-hour time

windows that were not followed by a sepsis event. Each panel represents different operationalized alarm thresholds for both SepsisFinder, GBDT and NEWS2. The alarm thresholds were chosen based on sensitivity (recall) matched to NEWS2 equal to 5 points (sensitivity 20%) and 7 points (sensitivity 42%) as well as SepsisFinder sensitivity 85%. Figure A includes all sepsis episodes. Figure B includes only hospital-onset sepsis episodes. The false alarms (green bars) are more or less randomly distributed across the 48h windows. The spikes at t=0 for the green plots is mostly due to the number of patients with short episodes that trigger an alarm with their first set of measurements. Note that the Y-axes are on different scales for each panel.

Abbreviations: SepsisFinder (SF), Gradient-boosting Decision Tree (GBDT), National Early Warning Score 2 (NEWS2), Hospital-onset (HO) and Hour (h).

Supplement Figure 3. Algorithm performance based on fixed time points in episodes where a sepsis event occurred in the validation data set

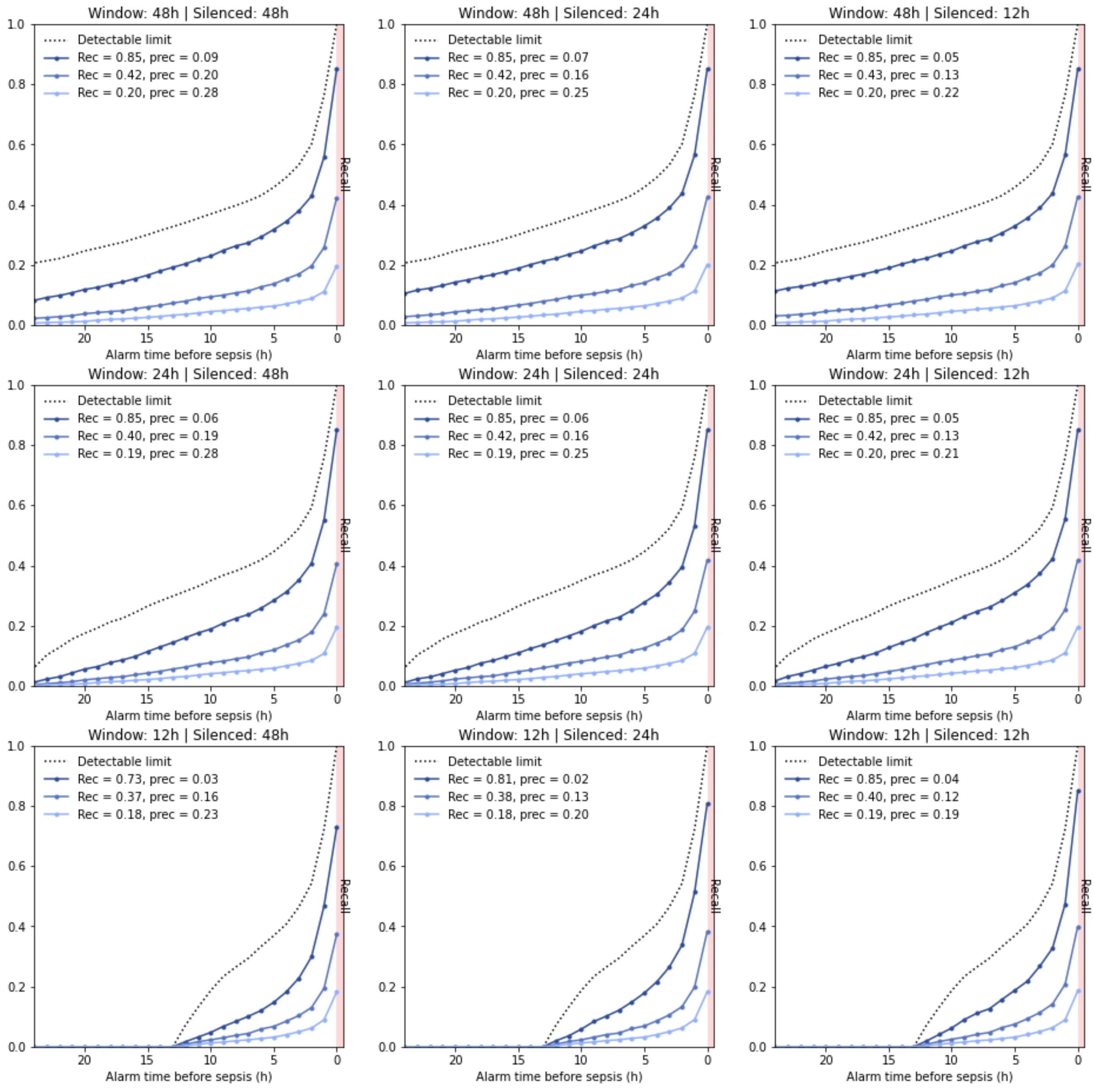

Legend: The figure shows the performance of the SepsisFinder model to predict sepsis at fixed time points before sepsis onset for three operationalized alarm thresholds. The alarm thresholds were chosen based on sensitivity (recall) matched to NEWS2 equal to 5 points (sensitivity 20%) and 7 points (sensitivity 42%) as well as sensitivity 85%. Since sepsis occurred at all times from admission to discharge, and predictions were only based on data from the current hospital episode, a dotted line has been added to represents the detectable limit for sepsis onset. The panels have been stratified based on the window in which alarms are considered true positives (12h, 24h, 48h), of the time for which alarms were silenced (12h, 24h, 48h). Precision (positive predictive value) decreased with shorter silencing and by shortening the window in which predictions can be considered true positive. The detectability also changed with the shorter windows.

Abbreviations: National Early Warning Score 2 (NEWS2), Recall (Rec), Precision (Prec) and Hours (h).
